# Fractional lattice charge transport

**DOI:** 10.1038/srep40860

**Published:** 2017-01-19

**Authors:** Sergej Flach, Ramaz Khomeriki

**Affiliations:** 1 Center for Theoretical Physics of Complex Systems, Institute for Basic Science, 34051 Daejeon, South Korea; 2 New Zealand Institute for Advanced Study, Center for Theoretical Chemistry & Physics, Massey University, 102904 Auckland, New Zealand; 3 Physics Department, Javakhishvili Tbilisi State University, 0128 Tbilisi, Georgia

## Abstract

We consider the dynamics of noninteracting quantum particles on a square lattice in the presence of a magnetic flux *α* and a dc electric field *E* oriented along the lattice diagonal. In general, the adiabatic dynamics will be characterized by Bloch oscillations in the electrical field direction and dispersive ballistic transport in the perpendicular direction. For rational values of *α* and a corresponding discrete set of values of *E*(*α*) vanishing gaps in the spectrum induce a fractionalization of the charge in the perpendicular direction - while left movers are still performing dispersive ballistic transport, the complementary fraction of right movers is propagating in a dispersionless relativistic manner in the opposite direction. Generalizations and the possible probing of the effect with atomic Bose-Einstein condensates and photonic networks are discussed. Zak phase of respective band associated with gap closing regime has been computed and it is found converging to *π*/2 value.

The two-dimensional electron gas in a perpendicular magnetic field is a celebrated topic in condensed matter physics (see e.g. ref. 1). Electron-electron correlations or electron-lattice interactions lead to fractional quantum Hall physics, while the integer quantum Hall effect is based on the properties of the single particle eigenstates in the presence of a weak dc electric field in the linear response regime. Underlying discrete lattice structures and symmetries can have substantial impact on the wavefunctions. The well-known case of a two-dimensional square lattice leads to the much-studied Harper model^2^ by reducing the two-dimensional problem to the dynamics in a one-dimensional quasi-periodic potential. The interplay of the two-dimensional lattice structure with magnetic fields *and* a substantial in-plane electric field is far from being well understood, despite some notable publications^3^,^4^ generalizing Hofstadter’s butterfly states to the case of applied electric fields and ref. 5, where asymmetric spreading regimes have been observed for few different directions of the in-plane electric field with respect to the lattice axes^6^.

Moreover, Kolovsky *et al*.^6^ observe intriguing small gap values in the quantum spectrum at particular values of the momentum *k* perpendicular to the dc field. The above studies miss a systematic tuning of the field strength which will be performed below. We stress that the square lattice is one of the most simple and therefore general periodic modulation of quantum dynamics in two dimensions. Applications in the field of photonics and ultracold atomic Bose-Einstein condensates are straightforward, at variance to the challenging studies of the dynamics of electrons due to the short lattice wavelength or dissipation in artifical superstructures.

The generalized translational invariance - with shifts in space and energy - is preserved in the presence of an electric field. A field orientation along a main lattice axis is the most simple yet trivial case since the shift is identical with the lattice spacing. A general orientation angle of the electric field relative to the lattice axes will lead to potentially very large or possibly even infinite shifts. We therefore focus on the most simple yet nontrivial case with the electric field being oriented along the diagonal of the square lattice, which leads to a period doubling of the shifts. The band structure is in general given by infinitely many interconnected bands gapped away from each other. The period doubling modifies the Wannier-Stark lattice at each given *k* into a bipartite one. We show here that the gaps between the bands surprisingly vanish at particular values of the electric field. Treatable cases correspond to rational relative magnetic flux values. The resulting band structure is given by intersecting left and right mover bands with opposite average group velocities. Moreover, the unexpected outcome is that the right movers have a relativistic linear dispersion. Wavepackets of initially localized particles are then shown to split into two parts, with a fractional relativistic current of right movers. While experiments using a two-dimensional electron gas might be a challenging task, our results could be directly verified in the context of Bose-Einstein condensates in optical lattices where the effective electric field is generated by a tilt of the lattice in the gravitational field^7^ or accelerating a whole lattice^8^, while the magnetic field is produced by artificial gauge fields^9^,^10^,^11^,^12^,^13^,^14^,^15^,^16^. Further, light propagation in waveguide networks can emulate the electric field analogy with a curved geometry of the waveguides^17^, while a special metallic fabrication of the waveguides and the surrounding medium^18^ leads to phase shifts of tunneling rates which leads to a magnetic field analogy. Alternatively, an artificial magnetic field can be achieved in dielectric waveguide lattices using an index gradient and periodic longitudinal modulations of the propagation constants^19^.

## The Model

**Figure 1:**
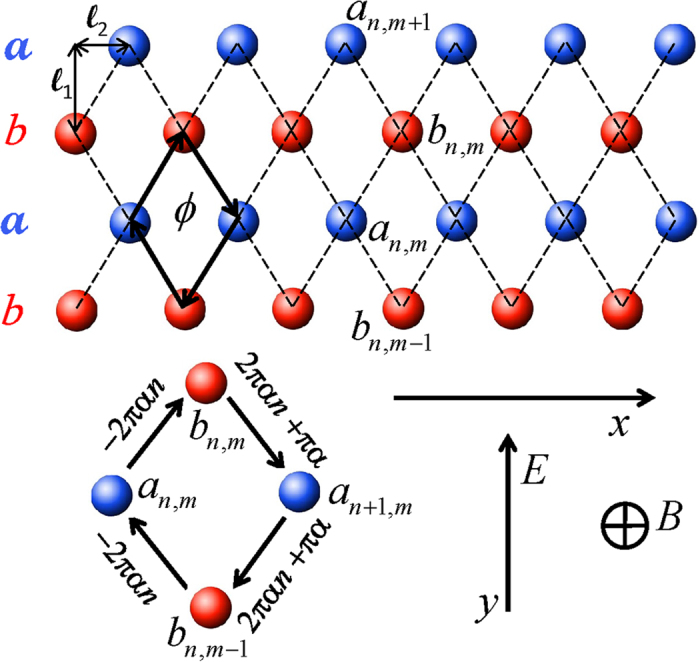
Schematics for the square lattice with legs *a* (blue) and *b* (red). Dashed lines connect sites with allowed hopping/tunneling of the particle. The magnetic flux *ϕ* is induced by the perpendicular magnetic field *B* and traverses the elementary rhombus with half diagonals 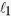 and 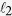. The corresponding phases *γ*_*ff′*_ are shown in the lower zoom of one plaquette, and the arrows indicate the direction of their integration. The dc electric field *E* is oriented along the *y*-axis. Therefore charge transport is observed along the *x* direction.

We consider the following Hamiltonian describing quantum particle dynamics in a tight-binding lattice (see Fig. 1), in the presence of out-off plane magnetic *B* ‖ *z* and in-plane electric *E* ‖ *y* dc fields:


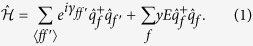


Here 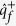 and 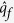 are standard particle creation and annihilation operators at the *f*-th lattice site and indexes *f* and *f′* denote two dimensional (*n*, *m*) lattice site positions. The first term in the Hamiltonian accounts for the hopping between nearest neighbor sites *f* and *f′* in the presence of a magnetic field. The corresponding phase factor is given^20^ by 
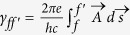
. The vector potential is defined in the Landau gauge as 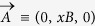. We denote the flux through the elementary rhombus (with diagonals 2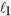 and 2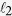) as 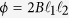. With the flux quantum *ϕ*_0_ = *hc*/*e*, the relative flux is defined as *α* = *ϕ*/*ϕ*_0_. The voltage drop between *a*, *b* chains follows as *V* = *El*_1_. The Schrödinger equation


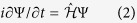


describes the evolution of the particle wave function Ψ with time-dependent complex probability amplitudes *a*_*n,m*_, *b*_*n,m*_ assigned to all lattice sites. With the notations *c*_*n*,2*m*_ = *a*_*n,m*_, *c*_*n*,2*m*+1_ = *b*_*n,m*_, *θ* = 2*πα* (flux angle) and the transformation


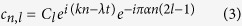


which takes the space direction *x* (index *n*) transversal to the applied electrical field into Fourier space with wave number *k*, we arrive at a simple one-dimensional bipartite chain (see Supplementary Information for derivation details)


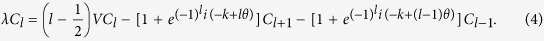


For each value of *k* the equation set (4) corresponds to a generalized bipartite Wannier-Stark ladder with a discrete unbounded spectrum *λ*_*ν*_(*k*). Bands with different indices *ν* will generically avoid intersections upon varying the wave number *k* due to level repulsion^21^. As a result, an initially localized electron will be trapped and perform generalized Bloch oscillations in the *y*-direction. At the same time, ballistic dispersive spreading will occur in the *x*-direction due to the overlap of the initial state with states from an effective finite number of bands. With 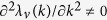 the spreading will be dispersive in both *x*-directions, since a whole spectrum of group velocities will lead to a widening of the wave packet.

**Figure 2:**
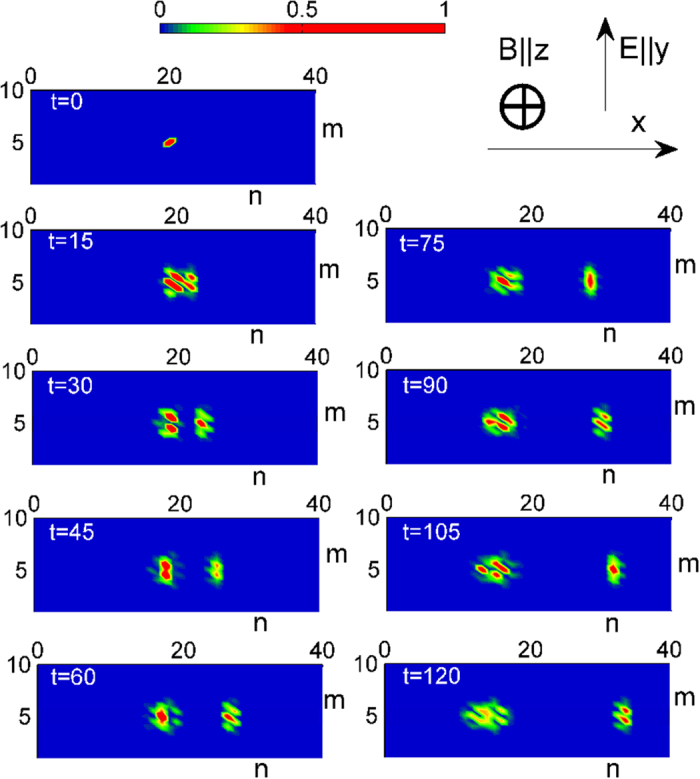
The evolution of the wave packet (|*a*_*n,m*_|^2^ and |*b*_*n,m*_|^2^) of a single electron placed initially in the center of the two-dimensional lattice. Distributions for different times are presented. The right top graph shows the orientation of the dc electric and magnetic fields.

We do observe this outcome in general, however, we find that for each value of relative magnetic flux *α* a value of the electric field exists, for which a localized fraction of the wave packet is propagating in the *opposite direction* with a well defined velocity *V*/(2*πα*). We also observe that precisely for those parameter values the gaps in the above discussed band structure vanish (this can indeed happen for matrices with elements depending on more than one parameter^21^). In Fig. 2 the wave packet is shown for *α* = 1/3 and 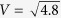 at different times. Indeed one third of the wave packet is propagating in a nondispersive localized manner to the right, while the complementary wave packet part is spreading as usual to the left (the wave packet dynamics is obtained by integrating equation (2) in time).

Clearly the condition for the occurence of a charge fractionalization must be routed in the zeroing of gaps in the band structure, which lead to effective left- and right-movers. To proceed we investigate the matrix 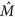 whose zero determinant is yielding the eigenvalues *λ* of (4):


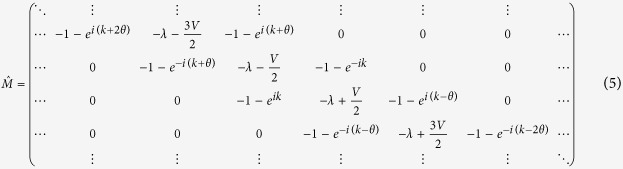


The band structure *λ*_*ν*_(*k*) follows from 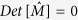. Matrix (5) is invariant under the symmetry operation


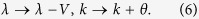


Further, the spectrum *λ*_*ν*_(*k*) is invariant under the symmetry operation


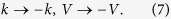


It follows, that if the eigenenergy *λ* is degenerated for some values of the voltage *V* and wavenumber *k*, then the eigenenergy *λ*′ = *λ* − *V* exists and is also degenerated for the same voltage *V* at wavenumber *k*′ = *k* + *θ*. Therefore closing one gap in the spectrum implies closing all symmetry related gaps as well. For the particular case of *λ* = 0 and *k* = *π* the matrix 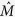 splits into two semi-infinite blocks, and we then arrive at the general statement that gaps must close for particular values of pairs of *θ* and *V* (see Supplementary Information for details).

## Results

**Figure 3:**
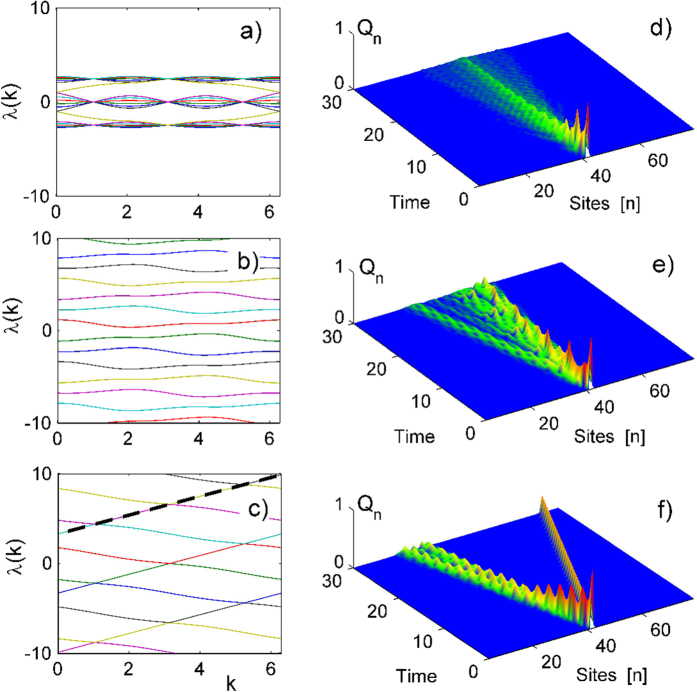
Graphs (**a**–**c**): Dispersion relations for the Bloch bands after diagonalizing matrix (5) with dimensions 80 × 80. The relative flux is *α* = 1/3 and the voltage drop take the values (from top to bottom) *V* = 0, *V* = 1 and 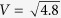. Graphs (**d**–**f**) show the wavepacket spreading upon integrating (2) with a single site initial condition using the parameters of the respective left panel graphs. The dynamics of the integrated charge density (9) accumulated in the *n*-th cross section is presented. The dashed line in graph (**c**) corresponds to the dispersionless curve 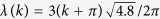.

Rigorous results are obtained for rational values of *α*. In these cases the matrix 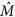 splits into noninteracting block matrices of finite size. Consider *α* = 1/3. The band structure is shown in Fig. 3(a–c) for various voltage drops. The matrix 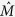 splits into 3 × 3 block matrixes, with one of them given by


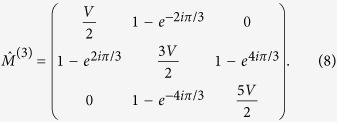


The condition 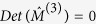 yields the roots *V* = 0 and 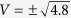. It follows that for relative flux *α* = 1/3 and voltage drop 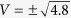 a nontrivial gap closing takes place which is indeed observed in Fig. 3(c). We then consider a particle initially localized on one site evolve this state in time according to (2). We compute the integrated charge density


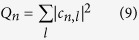


and plot the result in Fig. 3(d–f). While the cases *V* = 0, 1 yield the typical dispersive wave packet spreading, we find that for 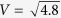 exactly one third of the wave packet is propagating in a relativistic nondispersive manner to the right, leading to the effect of charge separation (see Supplementary Information for a detailed calculation of the charge separation value). An analytical justification of this effect is straightforward observed in Fig. 3(c) where different bands are indicated by different colors. An initial single site excitation is a superposition of these bands, while each band contains Bloch modes with different wavenumber *k* and equal weight. Consider a specific band, e.g. the blue band: it has intersections with other bands separated by distance 2*π*/3 in *k*-space. Because of periodic boundary conditions in *k*-direction we can consider the bands on the cylinder (*λ*, *k*). One complete winding of the blue right mover band is balanced with two windings of the left mover band. Therefore precisely 1/3 of Bloch modes will have a positive group velocity and 2/3 of the Bloch modes will have negative group velocity. This explains the exact charge separation. The winding counting rule works for other values of relative flux *α* as well, with corresponding voltage drop *V* which ensure the gap closing regime.

For general rational relative flux values *α* = *p*/*s* one has to consider an *s* × *s* matrix 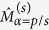. Then the condition 
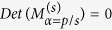
 gives *s* real roots for *V* = ±|*V*|. The root with the largest absolute value will yield *p*/*s* fractional transport, which can be found either analytically or numerically.

**Figure 4:**
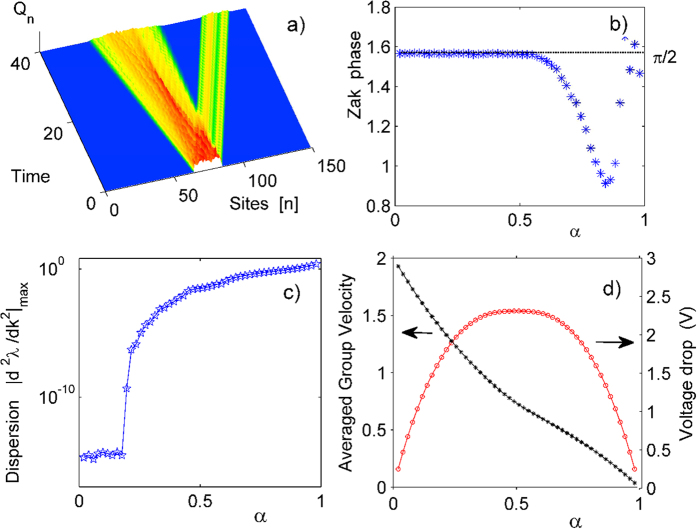
(**a**) Evolution of a wavepacket occupying 50 sites with initial random distribution of amplitudes and phases, for relative flux value *α* = 2/9 and voltage drop *V* = 1.8322. (**b**) The *α* dependence of the Zak phase (10). (**c**) The largest dispersion 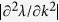 for the band with vanishing dispersion. (**d**) The *α* dependence of the largest voltage drop value *V*_1_ (red circles) and of the averaged group velocity 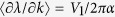 (black stars) for the analyzed cases of charge separation in the gap closing regime. Note that only rational values of *α* are considered here.

To check that the observed results are not an artifact of the specific choice of the initial state, in Fig. 4(a) we show the evolution of a broad initial wave packet which occupies 50 sites and has random amplitudes and phases, for *α* = 2/9 and the corresponding voltage drop *V* = 1.8322 (gap closing regime). We clearly observe dispersionless transport of a fraction of the total charge.

In Fig. 4(b) we plot the Zak phases of the right mover band with vanishing dispersion for various relative flux values and respective voltage drops corresponding to the gap closing regime. The Zak phase is computed as ref. 22


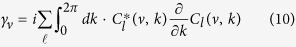


where *C*_*l*_(*ν*, *k*) is an eigenvector corresponding to the eigenvalue *ν* appearing from the diagonalization of (4). The Zak phase is usually a multiple of *π*, at least for one-dimensional lattices with inversion symmetry^22^, and is related to the existence of absence of edge states for open boundaries^23^. Opposite to that we find that the Zak phase takes the novel fractional value *π*/2 for *α* < 1/2. This might signal unusual edge physics - while the usual case *γ* = *π* relates to the existence of two edge states of an open chain, *γ* = *π*/2 might signal the existence of only one (right edge state in our case).

In the same Fig. 4 the largest dispersion 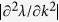 (c), and the averaged group velocity 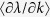 (d) for the analyzed cases of charge separation in the gap closing regime are presented. While the dispersion is very small but nonzero for *α* ≥ 1/6, it vanishes for *α* < 1/6. In the same limit of small *α* values the group velocities of the fractional charge tend to their largest values equal to 2. Also in this limit, the corresponding voltage drop *V* = 4*πα*.

So far we considered charge separation for the largest root of voltage drop given by 
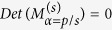
. Interestingly, the other roots yield an even more complex charge separation scenario. For *α* = 1/11 we examine the corresponding block matrix 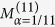 with dimension 11 × 11. The gaps close when the condition 
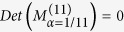
 is fulfilled which produces five nontrivial independent positive roots: *V*_5_ = 0.0869, *V*_4_ = 0.2244, *V*_3_ = 0.4135, *V*_2_ = 0.6585 and *V*_1_ = 0.9660.

The corresponding band structure and fractional charge dynamics with single site initial conditions are shown in Fig. S1. For the largest root *V* = *V*_1_ a fraction of 1/11 of the charge is separated and is propagating relativistically (see Supplementary Information for numerical evidence). This follows straight from the band structure, since only one of the eleven bands is yielding a positive nondispersive group velocity. The dependence of this largest voltage drop *V*_1_ on various rational values of *α* is shown in Fig. 4(d). We observe that in the limit of weak magnetic fields the corresponding voltage drop values tend to zero as well, however keeping the surprising feature of fractional relativistic transport.

For the other roots more bands are contributing to the fractional current, while still only one is completely gapless, as shown in Fig. S1. Moreover, the nonzero gaps become much smaller, leading to almost relativistic charge separation in both directions. The value of the charge fraction is now given by the number of the participating bands times the value of *α*. In our case of *V*_*m*_ nontrivial positive roots with *m* = 1, 2, 3, 4, 5 we find that the charge fraction is given by (2*m* − 1)*α* (see Supplementary Information for numerical evidence).

Up to now we have considered only rational values of relative flux *α*. The physics is the same for irrational values, i.e. for any fixed irrational *α* one can find a value of the voltage drop *V* for which the gap closing regime is realized. However, in order to compute the exact value of *V*, one has to consider infinite matrices, at variance to the finite rank *s* × *s* matrices for the case of rational *α* = *p*/*s*.

## Discussions

The observed fractional charge transport is crucially linked to the orientation of the electric field. In our case it is directed along the diagonal of a square lattice. Consider instead an electric field orientation along a main axis of the square lattice. In that case the momentum *k* of motion perpendicular to the electric field will completely decouple from the magnetic flux. As a result the matrix 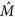 in Eq. (5) will not decouple anymore into two noninteracting blocks at a special value of *k*, since *k* will enter its diagonal part only. This will destroy the exact degeneracies which lead to fractional transport. Qualitatively the above findings can be also traced through a semiclassical approximation for weak magnetic fields similar to ref. 6. It would be worthwhile to analyze the connection of our results to non-Hermitian Hamiltonian dynamics as e.g. studied in ref. 24.

In summary, we demonstrate that a simple quadratic lattice is sufficient to obtain fractional charge separation of noninteracting electrons, or ultracold atomic gases, in the presence of magnetic fields, or synthetic gauge fields and a properly oriented and tuned DC bias. Such a charge separation can be potentially very useful for the preservation and engineering of entanglement in quantum systems. Potential technical difficulties such as Landau-Zener tunneling into higher orbitals, or scattering due to residual interactions and disorder, are known to be well controlable in the proposed setups of photonic networks and ultracold atomic condensates in optical potentials.

## Methods

We perform both analytical and numerical treatment of the initial model Hamiltonian (1). For analytical consideration we are seeking for eigenspectrum of the model (1) deducing it to matrix (5). Then using symmetry relations we find particular values of electric field for which fractional transport with vanishing dispersion is realized. While in numerical simulations we operate with evolution equation (2) taking single site occupation initial condition. Plugging the values of electric field obtained from analytical treatment we see fractional transport in numerical simulations.

## Additional Information

**How to cite this article**: Flach, S. and Khomeriki, R. Fractional lattice charge transport. *Sci. Rep.* **7**, 40860; doi: 10.1038/srep40860 (2017).

**Publisher's note:** Springer Nature remains neutral with regard to jurisdictional claims in published maps and institutional affiliations.

## Supplementary Material

Supplementary Dataset 1
