# Supplementary material for: Fractional lattice charge transport
Source: Sci Rep. 2017 Jan 19;7:40860. doi: 10.1038/srep40860 (PMC5244354; doi:10.1038/srep40860)
Supplement: Supplementary Dataset 1 [file srep40860-s1.pdf]

Supplemental Material for "Fractional lattice charge transport"

Sergej Flach[1,2], Ramaz Khomeriki[1,3],

[1] *Center for Theoretical Physics of Complex Systems, Institute for Basic Science, Daejeon, South Korea*

[2] *New Zealand Institute for Advanced Study, Centre for Theoretical Chemistry & Physics, Massey University, Auckland, New Zealand*

[3] *Physics Department, Tbilisi State University, Chavchavadze 3, 0128 Tbilisi, Georgia*

PACS numbers: 67.85.-d, 37.10.Jk, 03.65.Ge, 03.65.Aa

This Supplemental Material details 1) the derivation of the bipartite chain equation for an electron moving in a two-dimensional square lattice with a transverse magnetic field and a dc electric field oriented along the main diagonal of the square lattice (see main text for details of the definitions); 2) the general argument on the gap closing of the band structure; 3) the charge separation for multiple roots of gap-closing voltage drops 4) numerical results on charge current separation.

1. DERIVATION OF THE BIPARTITE CHAIN EQUATION

The Schrödinger equation for the wave function of a single quantum particle with Hamiltonian as defined in the main text, yields a set of differential equations for the wave function amplitudes $a_{n,m}$, $b_{n,m}$ on the a and b legs:

$$i\dot{a}_{n,m} = (2m-\frac{1}{2})Va_{n,m} - e^{2i\pi\alpha n}[b_{n,m}+e^{-i\pi\alpha}b_{n-1,m}] - e^{-2i\pi\alpha n}[b_{n,m-1}+e^{i\pi\alpha}b_{n-1,m-1}]\ , \qquad (1)$$

$$i\dot{b}_{n,m} = (2m+\frac{1}{2})Vb_{n,m} - e^{-2i\pi\alpha n}[a_{n,m}+e^{-i\pi\alpha}a_{n+1,m}] - e^{2i\pi\alpha n}[a_{n,m+1}+e^{i\pi\alpha}a_{n+1,m+1}]\ . \qquad (2)$$

We use the transformation

$$a_{n,m} = A_m e^{i(kn-\lambda t)}e^{-i\pi\alpha n(4m-1)}, \qquad b_{n,m} = B_m e^{i(kn-\lambda t)}e^{-i\pi\alpha n(4m+1)}\ , \qquad (3)$$

which takes the space direction x (index n) transversal to the applied electrical field into Fourier space with wave number k. The result is

$$\lambda A_m = (2m-\tfrac{1}{2})VA_m - [1+e^{i(-k+2m\theta)}]B_m - [1+e^{i(-k+(2m-1)\theta)}]B_{m-1}\ , \qquad (4)$$

$$\lambda B_m = (2m+\tfrac{1}{2})VB_m - [1+e^{i(k-2m\theta)}]A_m - [1+e^{i(k-(2m+1)\theta)}]A_{m+1}\ , \qquad (5)$$

with the flux angle $\theta = 2\pi\alpha$.

This result can be further simplified to a simple one-dimensional bipartite chain equation of the form

$$\lambda C_l = (l-\tfrac{1}{2})VC_l - [1+e^{(-1)^l i(-k+l\theta)}]C_{l+1} - [1+e^{(-1)^l i(-k+(l-1)\theta)}]C_{l-1} \qquad (6)$$

where $C_{2m} = A_m$ and $C_{2m+1} = B_m$.

2. ON THE GAP CLOSING IN THE BAND STRUCTURE

In order to identify the location of the energy level crossing (gap closing) points we note that for $k=\pi$ and $\lambda=0$ the matrix $\hat{M}$ splits into two semi-infinite matrixes

$$\hat{M}_1 = \begin{pmatrix} \ddots & \vdots & \vdots & \vdots & \vdots & \vdots \\ \cdots & 0 & 1-e^{-3i\theta} & -\frac{5V}{2} & 1-e^{-2i\theta} & 0 \\ \cdots & 0 & 0 & 1-e^{2i\theta} & -\frac{3V}{2} & 1-e^{i\theta} \\ \cdots & 0 & 0 & 0 & 1-e^{-i\theta} & -\frac{V}{2} \end{pmatrix}$$

and

$$\hat{M}_2 = \begin{pmatrix} \frac{V}{2} & 1-e^{-i\theta} & 0 & 0 & 0 & \cdots \\ 1-e^{i\theta} & \frac{3V}{2} & 1-e^{2i\theta} & 0 & 0 & \cdots \\ 0 & 1-e^{-2i\theta} & \frac{5V}{2} & 1-e^{-3i\theta} & 0 & \cdots \\ \vdots & \vdots & \vdots & \vdots & \vdots & \ddots \end{pmatrix}.$$

If $\lambda = 0$ is an eigenvalue, then $Det(\hat{M}) = 0$. At the same time $Det(\hat{M}) = Det(\hat{M}_1)Det(\hat{M}_2)$ and $\left|Det(\hat{M}_1)\right| = \left|Det(\hat{M}_2)\right|$. Then $Det(\hat{M}_1) = Det(\hat{M}_2) = 0$, and λ is two-fold degenerated for $\hat{M}$. In general we can expect that we can obtain a zero of the determinant of any of the two matrices $\hat{M}_1$ or $\hat{M}_2$ at a given flux angle θ upon proper tuning of the voltage drop V, since two independent ontrol paramaters are sufficient to close an avoided crossing completely.

3. ON CHARGE SEPARATION FOR MULTIPLE ROOTS OF GAP-CLOSING VOLTAGE DROPS

For $\alpha = 1/11$ we examine the corresponding block matrix $M^{(11)}_{\alpha=1/11}$ with dimension 11×11. The gaps close when the condition $Det\left(M^{(11)}_{\alpha=1/11}\right) = 0$ is fulfilled which produces five nontrivial independent positive roots: $V_5 = 0.0869$, $V_4 = 0.2244$, $V_3 = 0.4135$, $V_2 = 0.6585$ and $V_1 = 0.9660$.

The corresponding band structure (upper panel) and fractional charge dynamics (lower panel) with single site initial conditions are shown in Fig. 1. For the largest root $V = V_1$ a fraction of 1/11 of the charge is separated and is propagating relativistically (see next section for numerical evidence). This follows straight from the band structure, since only one of the eleven bands is yielding a positive nondispersive group velocity (see first column of Fig. 1).

For the other roots more bands are contributing to the fractional current, while still only one is completely gapless, as shown in Fig.1. Moreover, the nonzero gaps become much smaller, leading to almost relativistic charge separation in both directions (lower panels in Fig.1). The value of the charge fraction is now given by the number of the participating bands times the value of α. In our case of V_m nontrivial positive roots with $m = 1, 2, 3, 4, 5$ we find that the charge fraction is given by $(2m - 1)\alpha$ (see next section for numerical evidence).

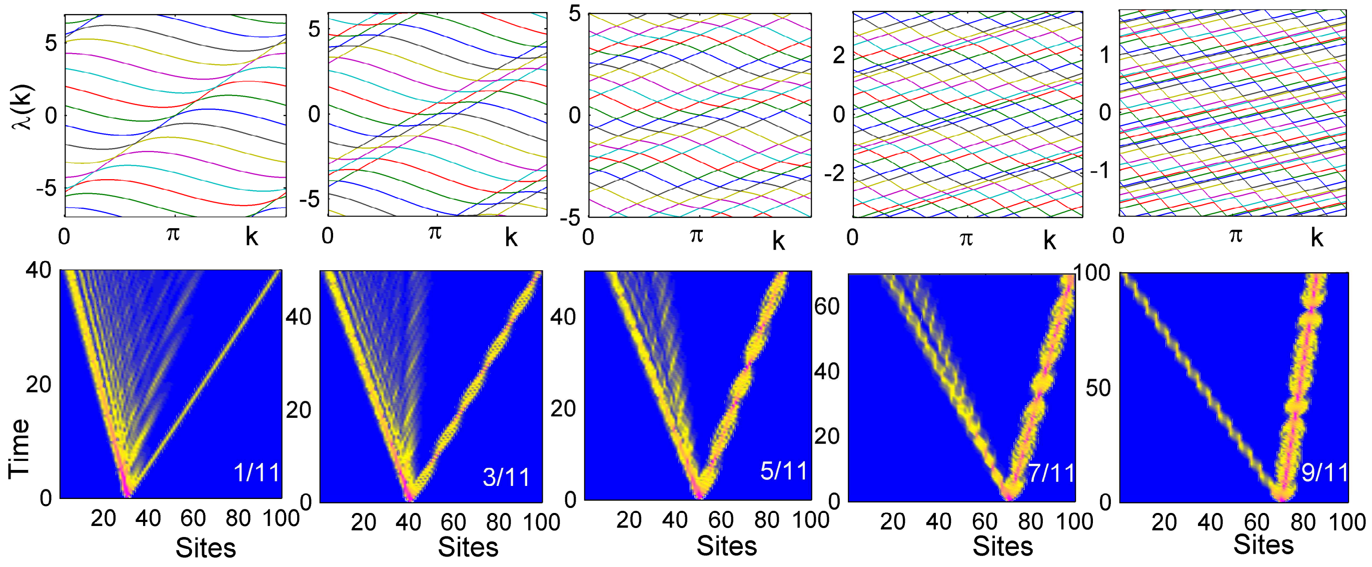

FIG. 1: Same as in Fig. 3 in the main text, but with different parameters: the relative magnetic field flux is $\alpha = 1/11$ and the voltage drop values are the five roots of the equation $Det\left(\hat{M}^{(11)}_{\alpha=1/11}\right) = 0$, in particular $V = 0.9660,\ 0.6585,\ 0.4135,\ 0.2244,\ 0.0869$ (from left to right). Note the different time scaling in lower panel.

4. ON THE NUMERICAL SIMULATIONS OF CHARGE CURRENT SEPARATION

With the definition

$$c_{n,l} = C_l e^{i(kn-\lambda t)} e^{-i\pi\alpha n(2l-1)} \tag{7}$$

and the integrated charge density

$$Q_n = \sum_l |c_{n,l}|^2 \tag{8}$$

we can compute the charge which is accumulated in a part of the lattice as

$$Q = \sum_{n=n_1}^{n_2} Q_n \ . \tag{9}$$

For the case $\alpha = 1/11$ we numerically integrate equations (1,2) on a square lattice with dimensions 100×100. We choose $n_1 = 80$ and $n_2 = 100$ and evaluate each of the five roots for the voltage drops in the gap closing regime (see main text for details). We compute the charge $Q(t)$. The results are plotted in Fig. 2 for the values $V_1 = 0.9660$, $V_2 = 0.6585$, $V_3 = 0.4135$, $V_4 = 0.2244$ and $V_5 = 0.0869$. Indeed $Q(t)$ saturates exactly at the values $1/11$ for $V = V_1$, while the saturation at values $3/11$, $5/11$, $7/11$ and $9/11$ is only approximate since not all the gaps close exactly for the corresponding voltage drop values.

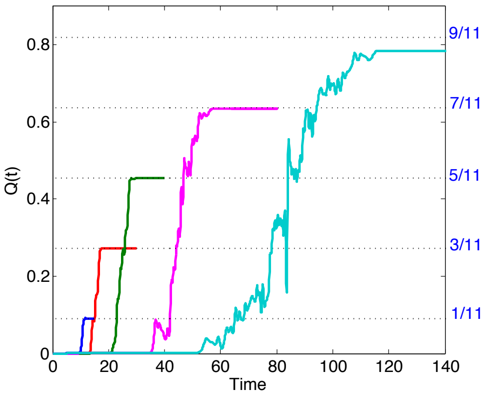

FIG. 2: The numerically calculated charge $Q(t)$ versus time for the relative flux value $\alpha = 1/11$ and five different voltage drop values $V_1 = 0.9660$, $V_2 = 0.6585$, $V_3 = 0.4135$, $V_4 = 0.2244$ and $V_5 = 0.0869$ saturating at the values $1/11$, $3/11$, $5/11$, $7/11$ and $9/11$, respectively. The lattice size is 100.
